# Interaction of copper potential metallodrugs with TMPRSS2: A comparative study of docking tools and its implications on COVID-19

**DOI:** 10.3389/fchem.2023.1128859

**Published:** 2023-01-26

**Authors:** Sergio Vazquez-Rodriguez, Diego Ramírez-Contreras, Lisset Noriega, Amalia García-García, Brenda L. Sánchez-Gaytán, Francisco J. Melendez, María Eugenia Castro, Walter Filgueira de Azevedo, Enrique González-Vergara

**Affiliations:** ^1^ Centro de Química del Instituto de Ciencias, Benemérita Universidad Autónoma de Puebla, Puebla, Mexico; ^2^ Laboratorio de Química Teórica, Depto. de Fisicoquímica, Facultad de Ciencias Químicas, Benemérita Universidad Autónoma de Puebla, Puebla, Mexico; ^3^ Departamento de Física Aplicada, Centro de Investigación y de Estudios Avanzados del Instituto Politécnico Nacional, Mérida, Mexico; ^4^ Departamento de Química Inorgánica, Facultad de Ciencias, Universidad de Granada, Granada, Spain; ^5^ Escola de Ciências da Saúde, Pontifícia Universidade Católica do Rio Grande do Sul (PUCRS), Porto Alegre, Rio Grande do Sul, Brazil

**Keywords:** TMPRSS2, COVID-19, molecular docking, Casiopeina-like metallodrugs, copper, DFT, Casiopeina analogs

## Abstract

SARS-CoV-2 is the virus responsible for the COVID-19 pandemic. For the virus to enter the host cell, its spike (S) protein binds to the ACE2 receptor, and the transmembrane protease serine 2 (TMPRSS2) cleaves the binding for the fusion. As part of the research on COVID-19 treatments, several Casiopeina-analogs presented here were looked at as TMPRSS2 inhibitors. Using the DFT and conceptual-DFT methods, it was found that the global reactivity indices of the optimized molecular structures of the inhibitors could be used to predict their pharmacological activity. In addition, molecular docking programs (AutoDock4, Molegro Virtual Docker, and GOLD) were used to find the best potential inhibitors by looking at how they interact with key amino acid residues (His296, Asp 345, and Ser441) in the catalytic triad. The results show that in many cases, at least one of the amino acids in the triad is involved in the interaction. In the best cases, Asp435 interacts with the terminal nitrogen atoms of the side chains in a similar way to inhibitors such as nafamostat, camostat, and gabexate. Since the copper compounds localize just above the catalytic triad, they could stop substrates from getting into it. The binding energies are in the range of other synthetic drugs already on the market. Because serine protease could be an excellent target to stop the virus from getting inside the cell, the analyzed complexes are an excellent place to start looking for new drugs to treat COVID-19.

## 1 Introduction

In December 2019, a new respiratory sickness called coronavirus disease 2019 (COVID-19) was found in Wuhan, China. Due to its rapid spread, COVID-19 was declared a global pandemic by the World Health Organization (WHO) on 11 March 2020. The causative virus was identified as the severe acute respiratory syndrome coronavirus 2 (SARS-CoV-2) (Hoffmann et al., 2020). Until December 2022, SARS-CoV-2 and its variants have almost infected 650 million people worldwide, and 6.6 million people have died as a result (Dong et al., 2020; World Health Organization, 2022). Even though effective drugs have not been found yet, the disease has been treated with antiviral and anti-inflammatory drugs, antibodies, corticosteroids, and plasma from people who have recovered from the disease (Ni et al., 2022).

The structure of SARS-CoV-2 has an external spike (S) glycoprotein that is needed to get into host cells. One of the methods for the virus to infect a cell (the other one is by endocytosis) begins with binding the viral S glycoprotein to the host receptor angiotensin-converting enzyme-2 (ACE2). Then, some viral glycoproteins go through activation by the transmembrane protease serine 2 (TMPRSS2), which proteolytically cleaves the binding for fusion between viral and host cells and allows membrane fusion and subsequent viral genome release (Wettstein et al., 2022). Although ACE2 is needed for SARS-CoV-2 infection, it may be hard to target it therapeutically because it plays a key role in metabolism, such as how the heart works. On the other hand, TMPRSS2 could be a more suitable target (Baughn et al., 2020). TMPRSS2 is expressed, depending on age, in epithelial cells of lung tissue, heart, liver, gastrointestinal tract, respiratory tract, prostate gland, and even the human corneal epithelium. It is involved in normal and abnormal processes like digestion, blood clotting, fertility, inflammatory responses, tumor growth, cell death, and pain (Thunders and Delahunt, 2020). In this way, TMPRSS2, which is found in human airways, helps activate important respiratory viruses like influenza and coronaviruses (Wettstein et al., 2022).

**FIGURE 1:**
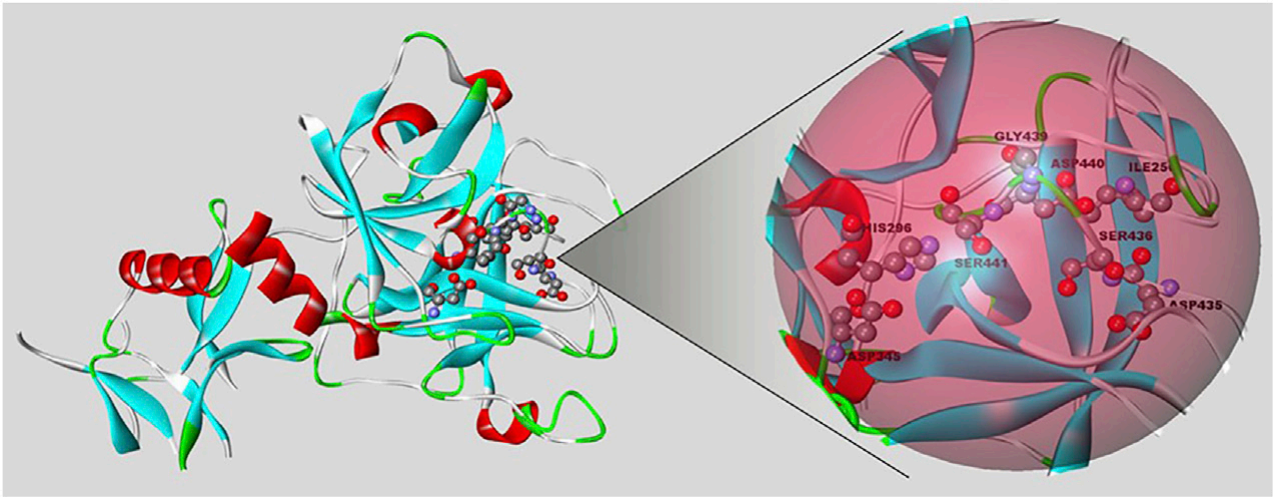
Representation of the TMPRSS2 protein (PDB code: 7MEQ) using Biovia/Discovery Studio v. 20.1. On the right side is the active site of the protein, where the triad of amino acids is located (His296, Asp345, Ser441).

The first published report about TMPRSS2 was in 1997, when its structure was described (Paoloni-Giacobino et al., 1997). This enzyme contains 492 amino acids, or 37 more in isoform 1 (Zmora et al., 2015), divided among an N-terminal intracellular domain, a hydrophobic transmembrane domain, and the stem region. The last one is made up of a low-density lipoprotein receptor class A domain (which is responsible for tethering the protease to the plasma membrane), a scavenger receptor cysteine-rich domain (that plays a role in protein-protein interactions and substrate recognition), and the C-terminal serine protease domain, which contains the amino acid triad essential for proteolytic activity (His296, Asp345, and Ser441) (Figure 1). The serine protease domain possesses the main proteolytic activity: It cleaves after Arg or Lys residues since it contains Asp435 at the base of the specificity pocket (S1 subsite) that binds to the substrate (Fraser et al., 2022). Because SARS-CoV-2 infection depends on TMPRSS2, protease inhibitors could be used to treat the disease. Some of the most studied serine protease inhibitors are camostat and nafamostat. Both inhibit the TMPRSS2 protease activity in human bronchial epithelial cells *in vitro*, but nafamostat shows higher efficiency.

Furthermore, studies *in vivo* in transgenic mice expressing the human *ACE2* gene show that nafamostat, delivered *via* intranasal, effectively reduces SARS-CoV-2 infection. Since nafamostat has been approved for decades as a treatment for other medical conditions, it could also be a good choice for treating COVID-19 (Li et al., 2021). In addition, some clinical studies on a few people have been done (Jang and Rhee, 2020; Takahashi et al., 2021), and although nafamostat appears to be effective against COVID-19, it could cause hyperkalemia and should be administered with heparin to compensate for its antifibrinolytic effect (Takahashi et al., 2021).

In searching for safe and effective drugs to treat the COVID-19 pandemic disease, metallodrugs, widely used in medicine, could be good candidates because coordination compounds have unique reactivity properties that cannot be achieved using only organic compounds (Cirri et al., 2021). Some metal ions, including selenium, iron, zinc, and copper, are known to block the interaction between the virus and the host cell, preventing the infection, inhibiting viral replication, destroying the viral structure, or inhibiting the activity of critical enzymes (Ni et al., 2022). In this regard, Casiopeinas^®^ are well-known planar copper(II) compounds with phenanthroline or bipyridine ligands. Even though these compounds are important because they fight cancer, parasites, and bacteria, new research has been done to explore their inhibitory effect against the main protease, M^pro^, which is responsible for the replication and primary transcription of the SARS-CoV-2 virus’s genetic material. It was concluded that most studied Casiopeinas^®^ could inhibit M^pro^ more efficiently than free monochelates, bioactive ligands, and boceprevir (a recognized inhibitor) (Reina et al., 2022).

Molecular docking is an *in silico* technique for determining the most stable configuration in which a specific molecule will connect to a receptor active site (Mhatre et al., 2021). Docking has become crucial in drug development, easing the burdensome process of finding functional therapeutic molecules (Tanveer et al., 2022). A scoring function that assigns a numerical fit value to a calculated protein/molecule configuration and a search algorithm that finds the molecule posture with the highest fit score in the protein binding site are the two critical components of every docking method (Halperin et al., 2002). Good docking is measured by two factors: The type of interaction and the docking score. Non-covalent bonds, Van der Waals interactions, π-stacking, and, in some cases, ionic bonds are examples of typical interactions (Mhatre et al., 2021).

Currently, there are only three approved drugs for COVID-19, Remdesivir, Molnupiravir, and Paxlovid, that inhibit viral replication (Gandhi et al., 2022; Jiang et al., 2022). However, several researchers have turned to drug repositioning to find quick and effective treatments. Also, molecular docking-based virtual screening seems to be a key way to find new antiviral drugs. Researchers can use this method as a different way to assign the synthesis of new compounds or the repositioning of drugs (Milite et al., 2019).

Following the research of drugs against SARS-CoV-2, in this work, seven Casiopeina analogs containing amino acids have been subjected to comparative *in silico* studies to determine their binding modes against the TMPRSS2 enzyme. Nafamostat and two Casiopeinas^®^, Cas III-ia (currently in clinical phase I trials in Mexico; Serment-Guerrero et al., 2011) and Cas IX-gly (Becco et al., 2014), have been used as comparative inhibitors.

## 2 Experimental section

### 2.1 Computational methods

The optimized molecular structures of the cationic complexes were calculated using the DFT method with the functional mPW1PW91 (Adamo and Barone, 1998) from the crystal structures already reported (Tovar-Tovar et al., 2004; Patra et al., 2009; García-Ramos et al., 2014; Martínez-Valencia et al., 2020; Rodrigues et al., 2020; Corona-Motolinia et al., 2021; Sánchez-Lara et al., 2021; Ramírez-Contreras et al., 2022) or modeled from them using Spartan'20 (Wavefunction Inc.). The basis set 6-311G(d) (Krishnan et al., 1980) was used for C, N, and O atoms, and 6-31G (Ditchfield et al., 1971) was used for H atoms. A valence double zeta with polarization on all atoms’ VDZP basis set (Wachters, 1970) was used for the Cu atom. These basis sets were used to achieve a well-balanced complete basis set. For aqueous solutions, the conductor-like polarizable continuum model (CPCM) (Cossi et al., 2003) was used to consider the solvent’s effect. The global reactivity indices, such as chemical potential (μ), electronegativity (χ), hardness (η), softness (s), and electrophilicity index (ω), were evaluated using the vertical Self-Consistent Field (ΔSCF) approach (Balawender and Geerlings, 2005). The vertical ionization potential (*I*) and the vertical electron affinity (*A*) were obtained from the energy difference between the ground state geometry and their corresponding ionized species from the optimized structures in an aqueous solution. All calculations were carried out in the Gaussian16 package (Frisch et al., 2016).

### 2.2 Molecular docking analysis

For molecular docking, copper(II) coordination compounds with bidentate ligands were used. The ligands are of type diimine (N, N), 2,2′-bipyridine, and (N, O) L-aminoacidatos of arginine, citrulline, asparagine, glycine, lysine, ornithine, glutamine, and theanine. Casiopeina III-ia and Casiopeina IX-gly were used for comparative purposes.

Three different molecular docking programs were employed to evaluate protein-complex interactions of the copper compounds with the transmembrane protease serine 2 (TMPRSS2): AutoDock4 (Morris et al., 2009), Molegro Virtual Docker (MVD) (Thomsen and Christensen, 2006), and GOLD software from the CCDC Mercury suite (Jones et al., 1997). To carry out the docking simulations, the protein TMPRSS2 with the protease inhibitor nafamostat (PDB code: 7MEQ) was used (Fraser et al., 2022). In addition, two sets of copper compounds were prepared, one with water molecules coordinated to copper(II) (named **System 1**) and the second one without water molecules (**System 2**). The nafamostat’s coordinates were taken out so that docking simulations for copper compounds could be done.

#### 2.2.1 Docking studies with AutoDock4

The docking process consists of two key steps; the first one is related to the conformation of the coordination complex and its orientation to the protein binding site, while the second key step consists of the prediction of the affinity of the complex to the protein using a scoring function.

To make a random search of the conformation of the copper complexes, the Lamarckian genetic algorithm was used. This algorithm considers the different complex poses and then interchanges between them, leading to a new generation of structures. Each member of the generation is evaluated with the scoring function, and only those values that meet the requirements (conformation, rotation, and orientation with respect to the protein) continue to the next-generation and so on until finding the best ligand conformations (Morris et al., 1998).

The force field used in AutoDock4 is a semiempirical free energy scoring function that considers the contribution of the hydrogen bonds and the electrostatic interactions. This scoring function discriminates the suitable poses from the wrong ones and estimates the affinity between the complex and the protein.

The protein and complexes were prepared through AutoDockTools4 by removing water molecules and polar hydrogens and adding Gasteiger charges. The receptor grid box was centered at x = 9.3, y = −5.9, and z = 19.993 Å. The box size was 40 Å^3^. Docking studies were done with 150 individuals in the population, a maximum energy evaluation of 2,500,000, and a maximum generation of 27,000 to result in 50 docking poses. The parameters for the copper(II) atom were the sum of the Van der Waals radii of two similar atoms (3.50 Å), the Van der Waals well depth (0.005 kmol mol^−1^), the atomic solvation volume (12.0 Å^3^), and the atomic solvation parameter (−0.00110). The hydrogen bond radius of the heteroatom in contact with hydrogen (0.0 Å), the well depth of the hydrogen bond (0.0 kcal mol^−1^), and various integers indicate the type of hydrogen bonding atom and indexes for the generation of the autogrid map (0, −1, −1, 1, respectively).

#### 2.2.2 Docking studies with molegro (MVD)

The MolDock scoring function implemented in MVD is the sum of the intermolecular energy ( $E_{inter}$ ) and the internal energy of the copper complex ( $E_{intra}$ ). The intermolecular interaction is calculated as follows:

$$E_{inter}=\underset{i}{\sum }\underset{j}{\sum }\left[332.0\frac{q_{i}q_{j}}{4r_{ij}^{2}}+PLP\left(r_{ij}\right)\right]$$

Subscripts *i* and *j* represent all the non-hydrogen atoms in the complex and protein. The first term is a Coulomb potential for charges *q*_*i*_ and *q*_*j*_. The variable *r*_*ij*_ represents the interatomic distance involving complex (*i*) and protein (*j*) atoms.

On the other hand, MVD defines intramolecular energy as follows:

$$E_{intra}=\underset{i}{\sum }\underset{j}{\sum }PLP\left(r_{ij}\right)+\underset{FB}{\sum }A\left[1-\cos\left(m\cdot \theta -\theta_{0}\right)\right]+E_{clash}$$

Summations are between all atom pairs in the complex except the atom pairs connected by two bonds or less. The term *FB* refers to the flexible bonds in the copper complex, and θ is the torsional angle of the bond. The last term ( $E_{clash}$ ) is a penalty of 1,000 applied if the distance between two atoms is less than 2.0 Å. The *PLP* is the piecewise linear potential in both equations. *PLP* uses two sets of parameters, one based on the Van der Waals interactions and the other for the hydrogen bonds (Thomsen and Christensen, 2006). Compared to other scoring functions, the MolDock score showed superior predictive performance (Thomsen and Christensen, 2006; Bitencourt-Ferreira and de Azevedo Jr., 2019).

The REDUCE program was employed for docking simulations with MVD to add hydrogens to the protein structure (Word et al., 1999). Atomic charges were assigned using the MVD program for all complexes and protein (Bitencourt-Ferreira and de Azevedo Jr., 2019). During docking simulation, the Ant Colony Optimization (Heberlé and de Azevedo Jr., 2011) search algorithm was combined with the MolDock scoring function (Thomsen and Christensen, 2006; Dias and de Azevedo Jr., 2008). To reproduce the results, 1123581321 was used as a random seed in all docking simulations, and the simulations were limited to a 12 Å radius sphere centered at the coordinates x = −9.17, y = −6.55, and z = 20.08 Å. After running docking simulations, the Nelder-Mead algorithm in MVD (Nelder and Mead, 1965) was used to find the protein-complex structures with the least energy.

#### 2.2.3 Docking studies with GOLD (genetic optimization for ligand docking)

The Goldscore function is a scoring function used to rank different ways of binding. It is based on molecular mechanics and has four terms:

GOLD Fitness = *Shb_ext + Svdw_ext + Shb_int + Svdw_int*

*Shb_ext* is the hydrogen-bond score between the protein and complex, and *Svdw_ext* is the Van der Waals score between them. *Shb_int* is the contribution to fitness from intramolecular hydrogen bonds in the complex. This term is turned off in all calculations (Verdonk et al., 2003) (this is the GOLD default and usually gives the best results). *Svdw_int* is the contribution from intramolecular strain in the complex. GOLD uses a genetic algorithm (GA) to change or improve parameters such as rotatable bonds, ring geometries, protein groups, and binding sites.

The Hermes software was used to carry out the protein preparation, which included removing water molecules before adding polar hydrogens and removing the nafamostat inhibitor. For the simulation, a maximum of 125,000 GA operations were carried out on a single population of 100 GA runs for each of the 10 independent GA runs. Crossover, mutation, and migration operator weights were left at their default values. The docking study was performed in the area comprising the active sites and the closest residues and constricted to a 10 Å radius sphere centered at the coordinates x = −6.04, y = −3.15, and z = 15.65 Å. The compounds were ranked by their GOLDscore.

## 3 Results and discussion

### 3.1 Global reactivity indices of the cationic complexes

**FIGURE 2:**
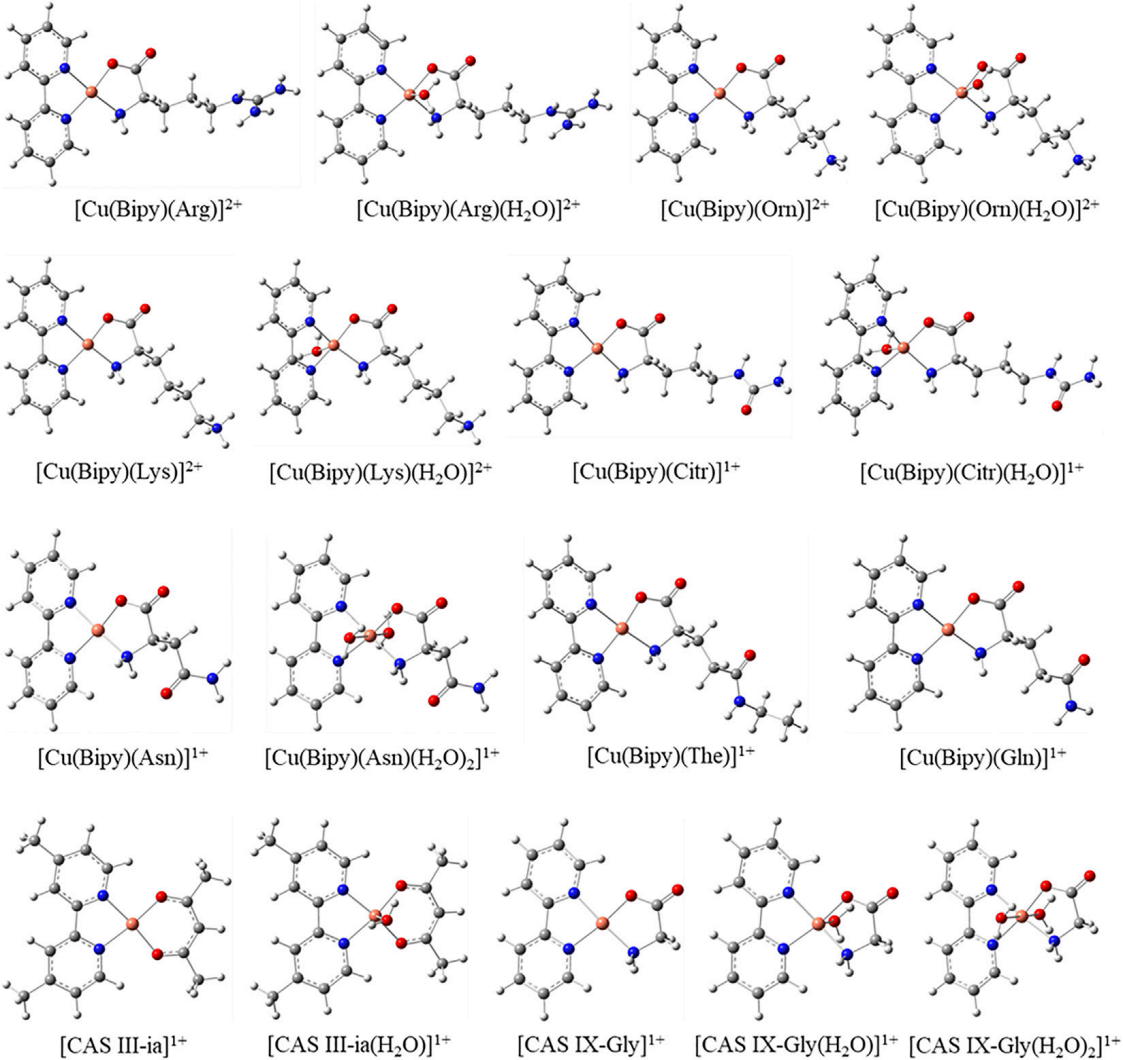
Optimized molecular structures of the copper complexes calculated with the mPW1PW91 functional in an aqueous solution.

Several reactivity indices have been analyzed to shed light on the structure-reactivity relationship of copper complexes. Firstly, in Figure 2, the optimized molecular structures for the seven complexes containing the amino acid residues Arg, Orn, Lys, Citr, Asn, The, Gln, and two Casiopeinas, Cas III-ia and Cas IX-Gly, containing acetylacetonato and Gly, respectively, are shown. Additionally, complexes containing Arg, Orn, Lys, Citr, Cas III-ia, and Cas IX-Gly have also been optimized with one water molecule in the apical position of Cu(II), while Asn and Cas IX-Gly also have two water molecules in both apical positions. In Table 1, the relevant parameters of the optimized molecular structures are compared with those reported crystal structures of compounds involving Bipy or Phen and aminoacidatos.

**TABLE 1:** Selected crystal structure and optimized parameters of the copper complexes calculated with the mPW1PW91 functional in an aqueous solution. Bond lengths in (Å) and bond angles in (°).

	Crystal structure				Calculated			
Complex	Cu−N	Cu−O	N−Cu−N	O−Cu−N	Cu−N	Cu−O	N−Cu−N	O−Cu−N
[Cu(Bipy)(Arg)]^2+^ Patra et al. (2009)	2.019^a^	1.939^c^	81.41^d^	91.10^f^	1.996^a^	1.914^c^	81.10^d^	92.87^f^
	2.038^a^		100.41^e^	83.63^g^	2.015^a^		102.79^e^	83.28^g^
	1.993^b^				2.014^b^			
[Cu(Bipy)(Orn)]^2+^ Martínez-Valencia et al. (2020)	1.997^a^	1.945^c^	80.59^d^	91.40^f^	1.994^a^	1.912^c^	81.20^d^	93.45^f^
	2.006^a^		99.52^e^	84.29^g^	2.013^a^		101.92^e^	83.82^g^
	2.001^b^				2.016^b^			
[Cu(Bipy)(Lys)]^2+^ Sánchez-Lara et al. (2021)	1.990^a^	1.936^c^	81.46^d^	91.41^f^	1.995^a^	1.911^c^	81.13^d^	93.04^f^
	2.002^a^		102.24^e^	83.57^g^	2.015^a^		102.24^e^	83.87^g^
	1.994^b^				2.014^b^			
[Cu(Bipy)(Citr)]^1+*^	2.001^a^	1.915^c^	82.31^d^	91.52^f^	1.996^a^	1.913^c^	81.06^d^	93.01^f^
Ramírez-Contreras et al. (2022)	2.008^a^		99.11^e^	85.93^g^	2.017^a^		102.72^e^	83.37^g^
	1.997^b^				2.011^b^			
[Cu(Bipy)(Asn)]^1+^	1.960^a^	1.980^c^	81.54^d^	92.95^f^	1.997^a^	1.919^c^	81.04^d^	93.09^f^
Rodrigues et al. (2020)	2.063^a^		102.59^e^	82.91^g^	2.014^a^		101.91^e^	83.98^g^
	1.976^b^				2.003^b^			
[Cu(Bipy)(The)]^1+^	—	—	—	—	1.996^a^	1.911^c^	81.07^d^	92.83^f^
					2.017^a^		102.49^e^	83.67^g^
					2.014^b^			
[Cu(Bipy)(Gln)]^1+^	2.005^a^	1.909^c^	81.78^d^	90.46^f^	1.996^a^	1.911^c^	81.07^d^	92.77^f^
Corona-Motolinia et al. (2021)	2.015^a^		101.25^e^	84.62^g^	2.016^a^		102.64^e^	83.69^g^
	2.018^b^				2.015^b^			
[Cas III-ia]^1+^ Tovar-Tovar et al. (2004)	1.973^a^	1.896^c^	81.43^d^	94.68^h^	2.000^a^	1.915^c^	80.93^d^	93.56^h^
	1.983^a^	1.885^c^			2.000^a^	1.915^c^		
[Cas IX-Gly]^1+^ García-Ramos et al. (2014)	1.992^a^	1.942^c^	81.22^d^	91.98^f^	1.995^a^	1.913^c^	81.08^d^	92.92^f^
	2.013^a^		99.80^e^	84.54^g^	2.016^a^		101.87^e^	84.34^g^
	2.003^b^				2.017^b^			

^a^ Cu–N bond length with N of Bipy or Phen.

^b^ Cu–N bond length with N of aminoacidato.

^c^ Cu–O bond length with O of aminoacidato.

^d^ N–Cu–N bond angle with both N of Bipy or Phen.

^e^ N–Cu–N bond angle with one N of Bipy or Phen, and one N of aminoacidato.

^f^ O–Cu–N bond angle with one N of Bipy or Phen, and one O of aminoacidato.

^g^ O–Cu–N bond angle with both N and O of aminoacidato.

^h^ O–Cu–O bond angle with both O of acetylacetonato for CAS III-ia complexes.

*The data corresponds to the D-citrullinato complex.

In most cases, the RMSD for these parameters are between 0.005 and 0.045 Å for the bond length, while the bond angles are between 0.69 and 2.42°. Furthermore, it indicates that the reliability of the DFT calculations is adequate, and the predicted geometrical parameters are a reliable source for predicting the chemical reactivity of the copper complexes.

In total, seventeen structures were calculated to analyze their global reactivity indices such as chemical potential ( μ ), electronegativity ( χ ), hardness ( η ), softness ( s ), electrophilicity index ( ω ), ang gap energy (E_gap_), that were obtained with the following equations:

$\mu =-\frac{\left(I+A\right)}{2}$ ; $\chi =\frac{\left(I+A\right)}{2}$ ; $\eta =\frac{\left(I-A\right)}{2}$ ; $s=\frac{1}{2\eta }$ ; $\omega =\frac{\mu^{2}}{2\eta }$ ; and $E_{gap}=I-A$ ; from the vertical ionization potential $I=E_{N+1}-E_{N}$ , and the vertical electron affinity $A=E_{N}-E_{N-1}$ , where $E_{N}$ is the electronic energy of the ground state, and $E_{N+1}$ and $E_{N-1}$ are the electronic energies of the system with one less electron and one more electron, respectively, according to the ΔSCF approach.

**FIGURE 3:**
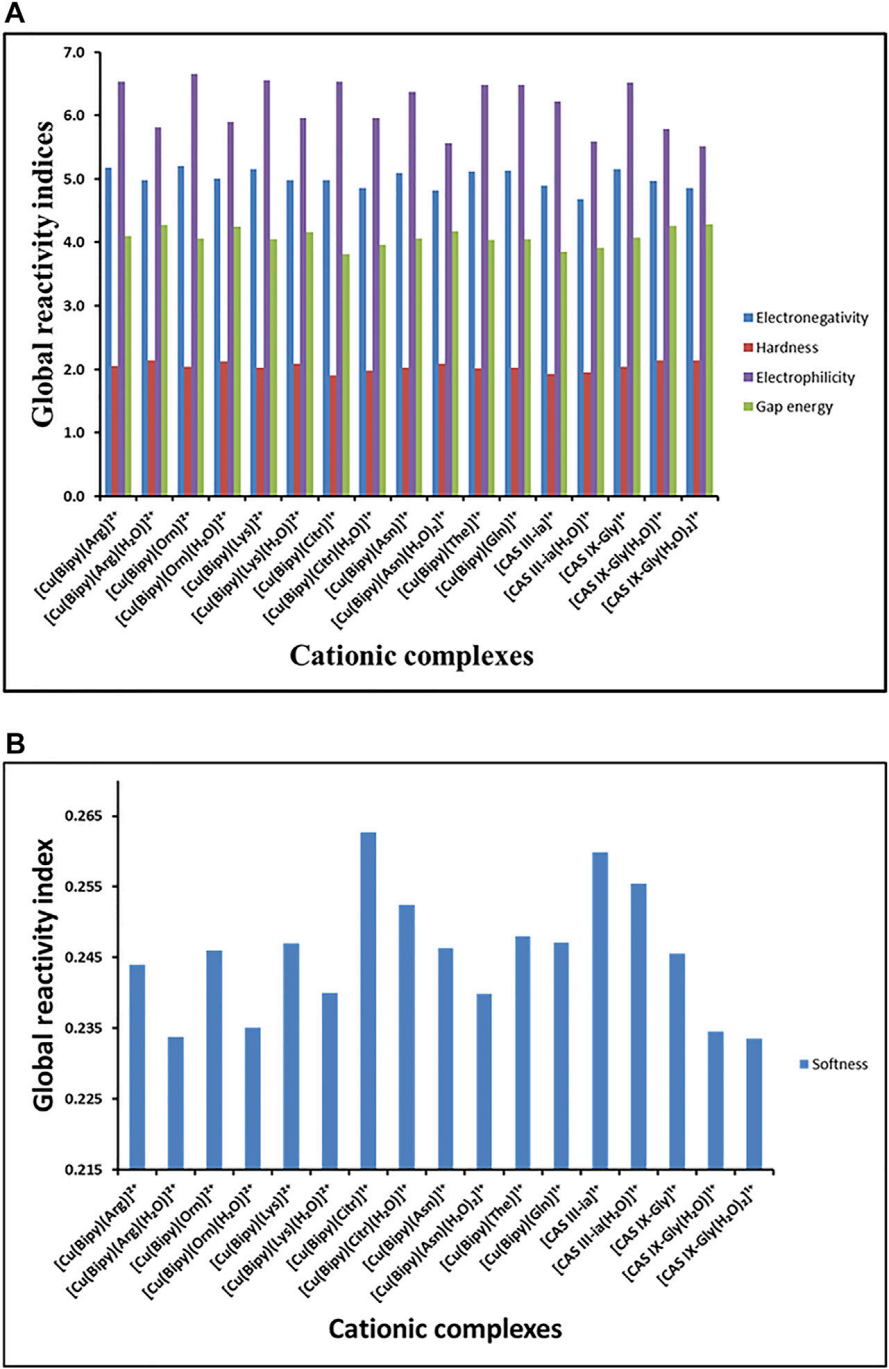
**(A)** Reactivity global indices χ ; η , ω , and E_gap_; and **(B)** Reactivity global index s of the copper complexes calculated with the mPW1PW91 functional in an aqueous solution.

The global reactivity indices for all compounds are collected in Supplementary Table S1; Figure 3. In Figure 3A the results show that the complexes with higher values of electronegativity χ (or lower chemical potential) are the copper complexes with Arg, Orn, Lys, The, Gln, and Gly, without water. These values of χ in the range of 5.11–5.20 eV indicate greater resistance to electron density loss or greater ability to attract electron density towards itself (Sert et al., 2014). Concerning the value of hardness η , [Cu(Bipy)(Arg)(H_2_O)]^2+^, [Cu(Bipy)(Orn)(H_2_O)]^2+^, [Cas IX-Gly(H_2_O)]^1+^, and [Cas IX-Gly(H_2_O)_2_]^2+^ are the hardest species. It means these complexes resist exchanging electron density with the environment and could be good nucleophiles. On the other hand, [Cu(Bipy)(Citr)]^1+^, [Cu(Bipy)(Citr)(H_2_O)]^1+^, [Cas III-ia]^1+^, and [Cas III-ia(H_2_O)]^1+^ have the smallest values of η ; thus they could be good electrophiles. Regarding the electrophilicity index, the complexes with values of 6.48–6.65 eV can also be considered good electrophiles, including the complexes with Arg, Orn, Lys, Citr, The, Gln, and Gly, without water. The gap energy values E_gap_, *i.e.*, the energy gained or lost in an electron donor-acceptor transfer, show that the most reactive complexes could be [Cu(Bipy)(Citr)]^1+^, [Cu(Bipy)(Citr)(H_2_O)]^1+^, [Cas III-ia]^1+^, and [Cas III-ia(H_2_O)]^1+^. Finally, in Figure 3B, it is possible to observe that the smallest softness values s , corresponding to [Cu(Bipy)(Arg)(H_2_O)]^2+^, [Cu(Bipy)(Orn)(H_2_O)]^2+^, [Cas IX-Gly(H_2_O)]^1+^, and [Cas IX-Gly(H_2_O)_2_]^1+^ could be the least toxic (Siddiqui and Javed, 2021).

### 3.2 Docking analysis

Molecular docking is a powerful tool for accelerating drug discovery to treat many diseases (Adelusi et al., 2022). For this reason, this technique was used to explore the possible interactions between TMPRSS2 and the copper compounds. To compare their results, three different docking programs were used (AutoDock4, Molegro Virtual Docker, and GOLD). The docked binding energies of the seven copper complexes, Cas III-ia and Cas IX-gly, with coordinated water molecules (**System 1**) and without them (**System 2**), along with the inhibitor nafamostat, are collected in Tables 2, 3.

**TABLE 2:** Results of docking simulations of **System 1**.

Compound	AutoDock (kcal·mol^−1^)	MolDock score (au)^a^	GOLDscore (au)^a^
[Cu(Bipy)(Arg)(H_2_O)]^2+^	−8.4	−140.881	54.792
[Cu(Bipy)(Orn)(H_2_O)]^2+^	−8.4	−135.100	55.903
[Cu(Bipy)(Lys)(H_2_O)]^2+^	−9.6	−133.086	55.124
[Cu(Bipy)(Citr)(H_2_O)]^1+^	−7.6	−124.847	51.513
[Cu(Bipy)(Asn)(H_2_O)]^1+^	−7.8	−117.995	45.626
[Cu(Bipy)(The)(H_2_O)]^1+^	−7.2	−127.699	51.685
[Cu(Bipy)(Gln)(H_2_O)]^1+^	−8.2	−111.124	49.352
[Cas III-ia(H_2_O)]^1+^	−7.3	−114.753	49.457
[Cas XI-Gly(H_2_O)]^1+^	−6.8	−99.1762	43.014
GBS^b^	−6.3	−87.8000	43.973

^a^ Arbitrary units (au).

^b^ Nafamostat.

**TABLE 3:** Results of docking simulations of **System 2**.

Compound	AutoDock (kcal·mol^−1^)	MolDock score (au)^a^	GOLDscore (au)^a^
[Cu(Bipy)(Arg)]^2+^	−8.3	−129.865	52.247
[Cu(Bipy)(Orn)]^2+^	−8.6	−129.439	56.716
[Cu(Bipy)(Lys)]^2+^	−8.6	−125.484	54.234
[Cu(Bipy)(Citr)]^1+^	−7.0	−125.296	49.403
[Cu(Bipy)(Asn)]^1+^	−8.2	−115.534	46.670
[Cu(Bipy)(The)]^1+^	−6.7	−121.692	51.717
[Cu(Bipy)(Gln)]^1+^	−7.7	−111.094	49.352
[Cas III-ia]^1+^	−6.4	−105.918	46.933
[CAS IX-Gly]^1+^	−5.8	−95.9585	41.827
GBS^b^	6.3	−87.8000	43.973

^a^ Arbitrary units (au).

^b^ Nafamostat.

Many potential metallodrugs have been investigated against different target proteins of SARS-CoV-2 (Karges and Cohen, 2021). Two proteases are considered the most essential for SARS-CoV-2 replication: The papain-like protease (PL^pro^) and the 3-chymotrypsin-like “main” protease (3CL^pro^ or M^pro^). This makes them attractive targets for potential therapies against COVID-19. Currently, some SARS-CoV-2 protease inhibitors are being studied, such as Lopinavir and Ritonavir, two already-approved Human Immunodeficiency Virus treatments (Tao et al., 2022). In this regard, coordination compounds have emerged as new candidates for PL^pro^ or M^pro^ inhibitors, including Zn(II) (DeLaney et al., 2021; Tao et al., 2022), Au(I) (Gil-Moles et al., 2020), Bi(III) (Yuan et al., 2020; Tao et al., 2021), Re(I) complexes (Karges et al., 2021), and other metals (Gandhimathi and Anbuselvi, 2022). Other inhibitory studies on the host receptor ACE2 with transition metal-based compounds (Al-Harbi, 2022) or against the spike protein with decavanadate (Favre et al., 2022) have also been conducted. Only a few compounds, however, have been studied against TMPRSS2, including some organic molecules such as nafamostat, camostat, and gabexate, all of them possessing a guanidinium group that interacts with Asp435 and an ester group pointing into Ser441 of the triad catalytic site, in a similar way to the small molecules here reported (Hu et al., 2021). In addition, polyoxotungstates have been examined, where [SiW_12_O_40_]^−4^ has a binding free energy of −9.4 kcal mol^−1^ toward the TMPRSS2, but none of the amino acids of the catalytic triad are present in the interactions (Shahabadi et al., 2022). Among coordination compounds, only two complexes based on Co(II) and Zn(II) have been studied, showing binding energies of −6.2 and −6.3 kcal mol^−1^, respectively, but again without interactions with the catalytic triad (Öztürkkan et al., 2022).

#### 3.2.1 Docking simulations with AutoDock4

**FIGURE 4:**
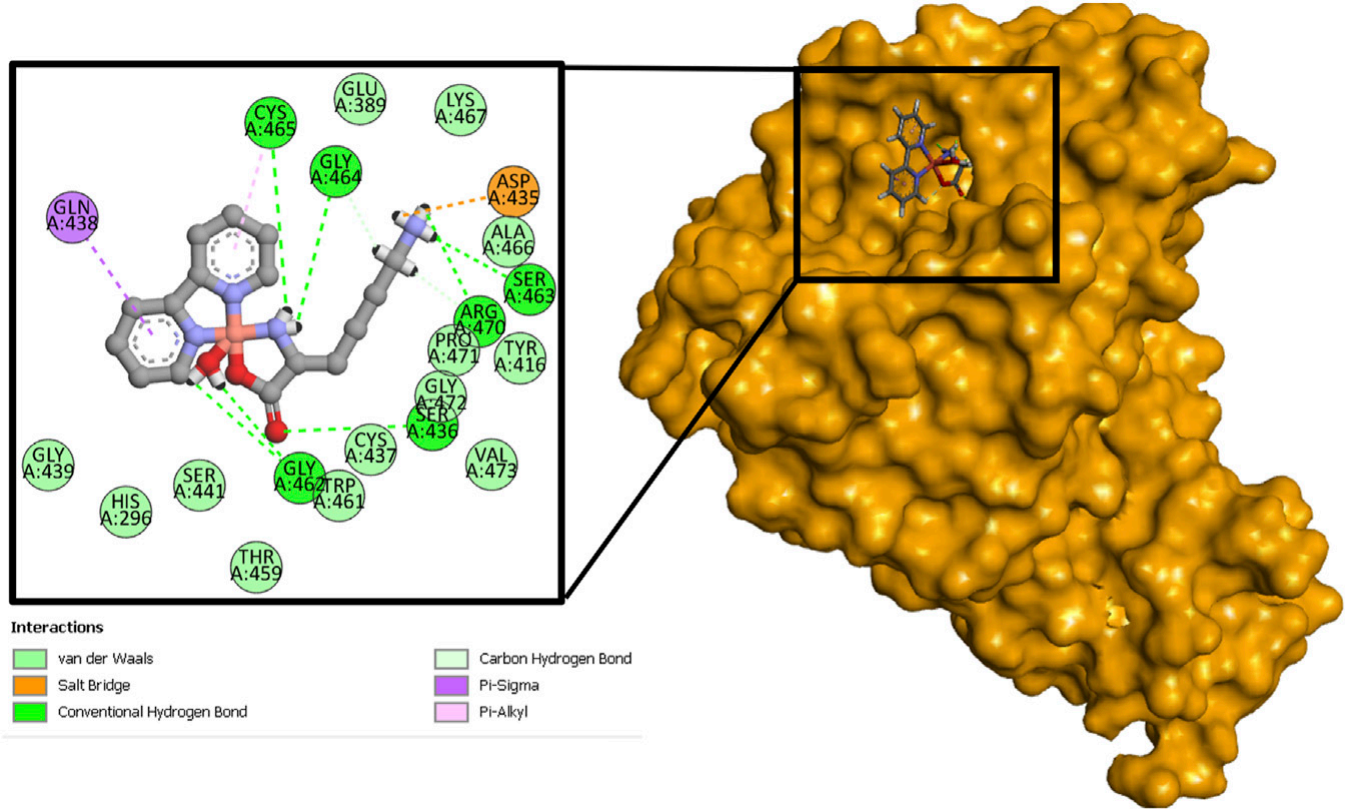
Binding interactions between [Cu(Bipy)(Lys)(H_2_O)]^2+^ and the protein (7MEQ).

The redocking results with the inhibitor nafamostat conserved the interactions with the amino acids of the catalytic site His296, Asp345, and Ser441. It presented an energy of −6.3 kcal mol^−1^. The compound that held the best binding free energy when compared to nafamostat and Casiopeinas^®^ was [Cu(Bipy)(Lys)]^2+^, followed by [Cu(Bipy)(Orn)]^2+^, and [Cu(Bipy)(Arg)]^2+^ in both Systems. The water molecule forms an extra hydrogen bond (Arg470), which is why there is a small increase in binding free energy when water is present in the complexes. Dicationic complexes of [Cu(Bipy)(Lys)(H_2_O)]^2+^, [Cu(Bipy)(Orn)(H_2_O)]^2+^, and [Cu(Bipy)(Arg)(H_2_O)]^2+^ form several hydrogen bonds that include the amino acids Ser436 and Gly464, and one salt bridge with Asp435. Although they do not present hydrogen bonds with the triad of interest, they present hydrophobic interactions with His296 and Ser441 (Figure 4).

The results obtained with AutoDock4 for **Systems 1**, **2** are found to be similar in the complexes with Arg, whereas for the complexes [Cu(Bipy)(Orn)(H_2_O)]^2+^ and [Cu(Bipy)(Lys)(H_2_O)]^2+^ the presence of the water molecule slightly changes the disposition of the complexes when they are interacting with the protein. Furthermore, this water molecule interacts with other amino acids of the protein, which explains the changes in binding free energies found in these complexes.

The binding affinity of [Cu(Bipy)(Lys)]^2+^ with AutoDock is similar to that shown by a Cu(II)-phenanthroline compound against M^pro^ (−9.0 kcal mol^−1^) (Aprajita and Choudhary, 2022). In addition, when compared to previous docking analyses of anti-SARS-CoV-2 drugs like remdesivir (−6.77 kcal mol^−1^), chloroquine (−6.93 kcal mol^−1^), and dexamethasone (−7.77 kcal mol^−1^), all the calculated binding affinities are relatively higher (Shivanika et al., 2022).

#### 3.2.2 Docking simulations with MVD

**FIGURE 5:**
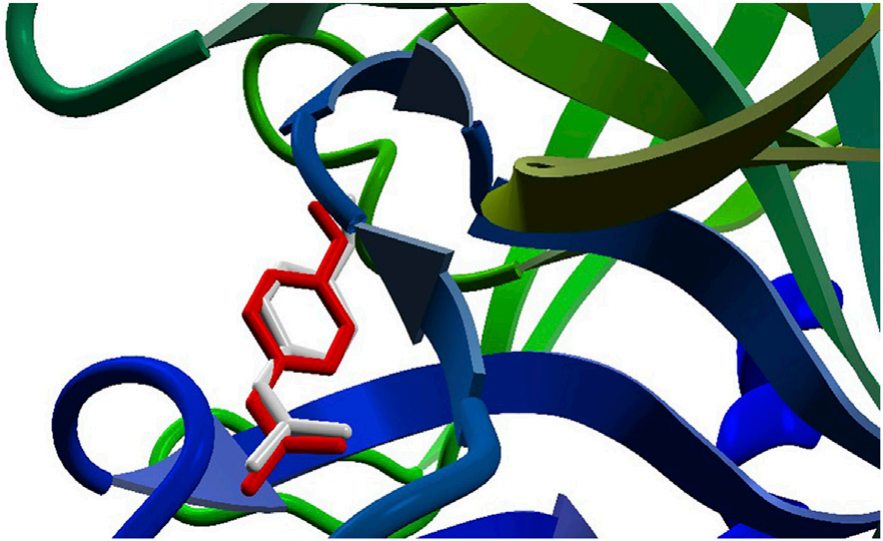
The re-docking result of the structure 7MEQ. MVD generated an RMSD of 0.58 Å. The pose structure of the nafamostat is indicated in red, whereas the inhibitor’s crystallographic coordinates are light gray—an image generated by the MVD program.

**FIGURE 6:**
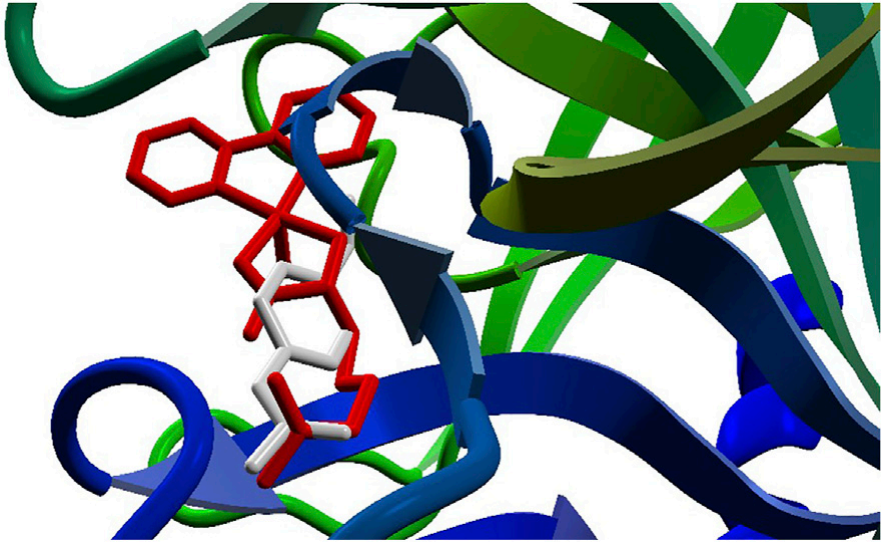
Docking results for the compounds [Cu(Bipy)(Arg)(H_2_O)]^2+^ (red) and nafamostat (light gray).

**FIGURE 7:**
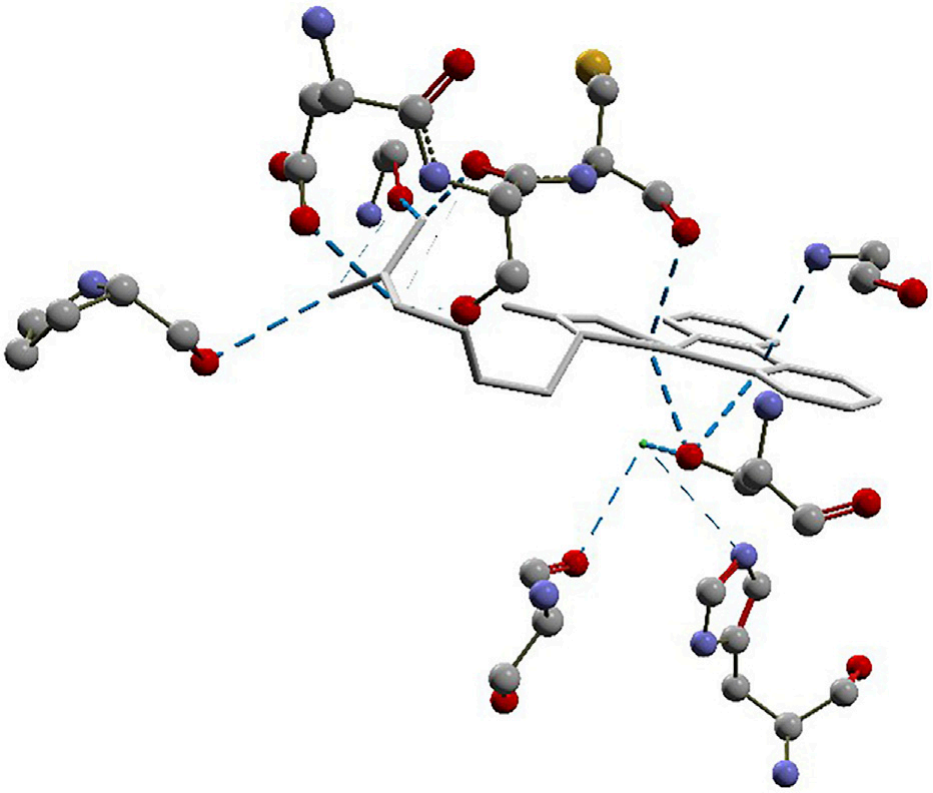
Intermolecular hydrogen bonds (dashed blue lines) between the [Cu(Bipy)(Arg)(H_2_O)]^2+^ compound and the protein.

The docking approach is validated with the atomic coordinates of the structure 7MEQ. The lowest energy pose generated a docking root-mean-squared deviation (RMSD) of 0.58 Å, as shown in Figure 5. Docking simulations of the structures of **System 1** pointed out the copper compound [Cu(Bipy)(Arg)(H_2_O)]^2+^ with the lowest binding energy (Figure 6), lower than nafamostat and Casiopeinas^®^. Analysis of this compound’s intermolecular interactions (Figure 7) indicates 14 hydrogen bonds, three involving the water coordinating the Cu(II). The following residues in the hydrogen bonds were found: His296, Asp434, Ser436, Cys437, Gly439, Ser441, Ser460, Gly464, and Pro471. Analysis of the intermolecular hydrogen bonds for the complex involving nafamostat shows the conservation of the interactions involving the following amino acids: Asp435, Ser436, Gly439, Ser441, and Gly464. The overall network of hydrogen bonds is conserved in the structure [Cu(Bipy)(Arg)(H_2_O)]^2+^. Only residues His296, Cys437, and Pro471 are specific for the copper compound. These additional interactions observed for [Cu(Bipy)(Arg)(H_2_O)]^2+^ contribute to the lowest energy determined for the complex.

Docking simulations for **System 2** revealed [Cu(Bipy)(Arg)]^2+^ to be the lowest energy complex. Although this complex has no water molecule coordinating with the copper, most intermolecular interactions are conserved.

#### 3.2.3 Docking simulations with GOLD

**FIGURE 8:**
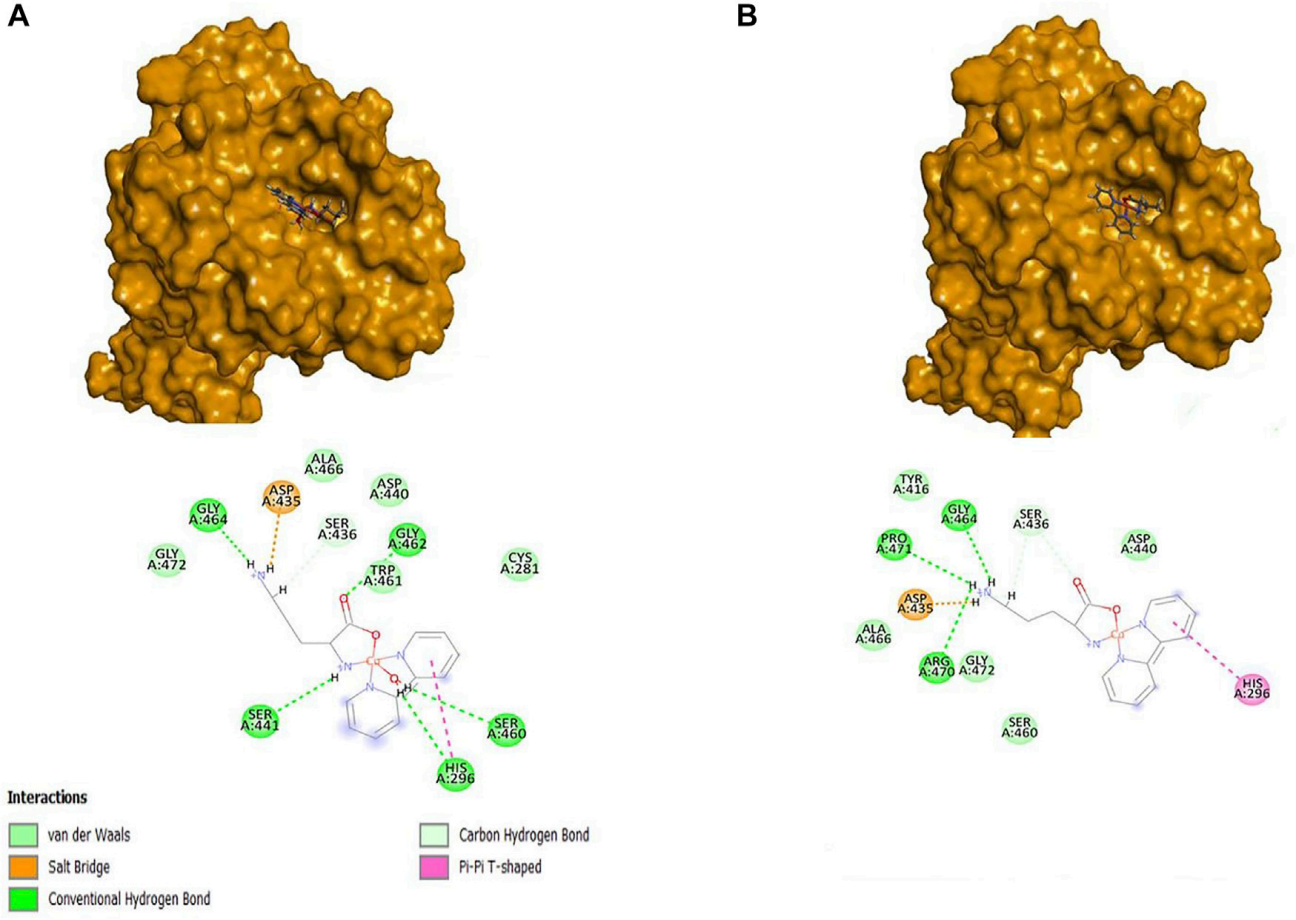
Representations 3D and 2D of the [Cu(Bipy)(Orn)(H_2_O)]^2+^ **(A)** and [Cu(Bipy)(Orn)]^2+^ **(B)** with 7MEQ.

For both Systems, the Orn compound was the one that obtained the highest score, followed by the complexes of Lys and Arg, compared to nafamostat and Casiopeinas^®^. The compound [Cu(Bipy)(Orn)(H_2_O)]^2+^ formed a total of six hydrogen bonds with His296 in addition to Ser436, Ser441, Ser460, Gly462, and Gly464. Also, this complex formed a salt bridge with Asp435, a π-π interaction with His296, a π-bond with His296, and hydrogen bonds with Pro471, Gly464, and Ser436 residues, as shown in Figure 8, as well as a salt bridge with Asp435. On the other hand, [Cu(Bipy)(Orn)]^2+^ formed five hydrogen bonds. In both Systems, there are interactions with the residues of the triad; in the case of **System 1**, water coordination helps to form hydrogen bonds with the residues of amino acids, contributing to the final ligand interaction.

The Lysine compound was discovered to form four hydrogen bonds with the residues Gly464, Pro471, Ser441, and Ser436, as well as a salt bridge with Asp435 and a π-sulfide interaction with Cys281. The water, in this case, had no interaction with any amino acid residue. In **System 2**, the lysine complex formed three hydrogen bonds with the amino acids Gly464, Gly439, and Ser 436, as well as a salt bridge with Asp435, a π-π interaction with His296, and a π-alkyl interaction with Cys281.

For the Arginine compound, eleven hydrogen bonds were observed with the residues of Asp435, Gly464, Ser460, Gly439, Ser441, Asp440, Gly462, and Ser436, a π-π interaction with His296, and a π-alkyl with Cys281 for the **System 1**. For **System 2**, the Arginine compound presented ten hydrogen bonds with the residues Asp435, Gly462, Ser436, Asp440, Ser441, Gly439, Ser460, and Gly464, a π-π interaction with His296, and a π-alkyl with Cys281. In both compounds (Lysine and Arginine) in **System 1**, it was not observed that the water coordinate had some interaction with some residue; however, in both **Systems**, bonds were formed with the amino acids of the catalytic triad.

Finally, the correlation between the scores (binding energies) calculated using MolDock and those determined using GOLDscore and AutoDock4 was also analyzed. We have a positive Pearson correlation of 0.761 between MolDock and AutoDock4 for both **Systems**. The correlation between the MolDock score and the GOLDscore is −0.911 and −0.878 for **Systems 1** and **2**, respectively. There is a negative correlation since GOLDscore assigns the highest values for the best hits. For all scoring functions, the three best hits found in **System 1** are the compounds [Cu(Bipy)(Arg)(H_2_O)]^2+^, [Cu(Bipy)(Orn)(H_2_O)]^2+^, and [Cu(Bipy)(Lys)(H_2_O)]^2+^, and for **System 2**, all three scoring functions identified the same three best compounds with differences in the order: [Cu(Bipy)(Arg)]^2+^, [Cu(Bipy)(Orn)]^2+^, and [Cu(Bipy)(Lys)]^2+^.

COVID-19 still stresses healthcare systems and causes a high mortality rate worldwide 3 years after the outbreak. Remdesivir, Paxlovid, and molnupiravir, three oral antivirals, have been licensed in several countries. However, the best treatment option is still required, so new drugs and novel uses for current ones are expected in 2023. People who cannot access vaccines, whose immune systems do not fully respond to immunization, or who develop intercurrent infections need new medicines. Furthermore, Philippe Guérin, head of the Oxford University Infectious Diseases Data Observatory, pointed out that many clinical trials focus on therapies that would be too expensive or difficult to use in many countries, creating a division between research and low- and middle-income nations (Ledford, 2022). Currently, most efforts are focused on antibodies, organic molecules, or already approved drugs for other diseases, such as chloroquine, favipiravir, remdesivir, molnupiravir, nirmatrevir, paxlovid (Akhtar, 2020), or the M^pro^ inhibitor in phase 3 trial, S-217622 (ensitrelvir) (Sasaki et al., 2022). As an alternative, metal-based compounds have also been explored as anti-SARS-CoV-2 agents (Cirri et al., 2021; Gil-Moles et al., 2021; Karges and Cohen, 2021; Vlasiou and Pafti, 2021; Li et al., 2022; Ni et al., 2022).

Coronaviruses and influenza viruses rely heavily on TMPRSS2 for host entry and dissemination (Stopsack et al., 2020; Wettstein et al., 2022). This includes SARS-CoV, the agent responsible for the 2003 SARS outbreak, and influenza H1N1, the virus responsible for the 1918 and 2009 influenza pandemics (Chaipan et al., 2009; Matsuyama et al., 2010; Hoffmann et al., 2020). These examples illustrate the central and conserved function of TMPRSS2 in the pathogenesis of diseases caused by coronaviruses and influenza viruses. The inhibitor, Camostat mesylate, partially prevented the entry of SARS-CoV-2 into lung epithelial cells in an *in vitro* investigation involving cell lines and primary pulmonary cells (Hoffmann et al., 2020). In a TMPRSS2 deletion model, mice infected with the H1N1 influenza virus exhibited a significantly reduced illness course, with protection from pulmonary pathology, weight loss, and death, compared to wild-type control mice (Hatesuer et al., 2013). Given its prominent role in beginning SARS-CoV-2 and other respiratory viral infections, it is believed that regulating TMPRSS2 expression or activity represents a suitable target for prospective COVID-19 treatments. Key functional residues of TMPRSS2 (His296, Ser441, and Ser460) interacted with nearby residues of SARS-CoV-2 spike protein cleavage sites. The TMPRSS2 region interacts with the C-terminal cleavage site (Arg815/Ser816) of the SARS-CoV-2 spike protein. This site was considered more druggable than the N-terminal cleavage site (Arg685/Ser686). Therefore, a complex made up of human TMPRSS2 and SARS-CoV-2 spike protein is suggested as a potential drug target that could be used to guide structure-based drug design (Hussain et al., 2020).

Molecular docking has demonstrated that copper(II) complexes can interact with crucial SARS–CoV–2 targets such as M^pro^, PL^pro^, spike protein, and ACE2 (Al-Harbi, 2022; Viola et al., 2022). Among them, the square planar complex [Cu(L)_2_], where L = 2-(4-morpholinobenzylideneamino)phenol (Sakthikumar et al., 2022), shows binding energy of −7.8 kcal mol^−1^ against M^pro^ with Autodock Vina (interactions are not specified), higher than the results of the copper complexes with Arg, Orn, and Lys, here studied. In addition, fifty Casiopeinas^®^ and related Cu(II) compounds were also investigated as M^pro^ inhibitors with AutoDock (Reina et al., 2022). Some Casiopeinas^®^, such as CasII-5Clsa, CasII-ambz, or CasII-tyr, show promising results with binding energies between −8.58 and −9.25 and kcal·mol^−1^, lower than the references boceprevir and N3 peptide, and they interact with His41, Asn142, Cys145, Glu166, and Gln189, which are part of the catalytic site cavity of M^pro^ (Kneller et al., 2020). However, as TMPRSS2 inhibitors, Cas III-ia and Cas IX-gly exhibit high binding energies with values between −5.8 and −7.3 kcal mol^−1^.

Here we have shown that copper(II) complexes derived from amino acids, analogs of Casiopeinas^®^, could be considered good candidates for potential metallodrugs against COVID-19, as compared with Casiopeinas^®^ already in phase I clinical trials and nafamostat. The cationic nature of the analogs and the basic terminal nature of the side chains of the amino acids are responsible for anchoring them close to the active site by interacting with Asp495, a key amino acid residue for interacting with arginine or lysine residues of target proteins.

## 4 Conclusion

The optimized molecular structures of seven complexes containing the amino acid residues: Arg, Orn, Lys, Citr, Asn, The, and Gln; and two Casiopeinas: Cas III-ia and Cas IX-gly, containing acetylacetonato and Gly, respectively, were investigated using DFT methodology, and the global reactivity indices were determined. The highest gap energy values between 4.17 and 4.28 eV suggest that the complexes with Arg, Orn, and Lys with a molecule of water and for Asn with two molecules of water are the most stable and can present bioactivity, with comparable values to Casiopeinas^®^. Additionally, the softness index appeared to have the smallest values between 0.234–0.240 eV for the same complexes with Arg, Orn, and Lys with a molecule of water and Asn with two molecules of water, comparable with the values for CAS IX-Gly with one and two water molecules (0.234 and 0.235 eV, respectively). A low value of the softness index is related to low toxicity through electrophilic-nucleophilic interactions, and it can be used as a descriptor of their biological activity with the TPMRSS2 protein.

AutoDock4, MVD, and GOLD have different scoring functions and search algorithms to carry out docking simulations. Nevertheless, they identified the same top three compounds for both systems, indicating the convergence of our docking approaches. Also, in the three Docking methodologies, the following similarities were found: i) An improvement in the binding energy/score when only one water molecule is in the structure of the studied complexes; ii) the binding energy/docking score is better for the studied complexes than for the nafomastat inhibitor; and iii) the compounds that interact best with the protein are the complexes with the amino acid residues Orn, Lys, and Arg, though the order of these amino acid residues varies between them.

Since the copper compounds localize just above the catalytic triad, they could stop substrates from getting into it. The binding energies are in the range of other expensive synthetic drugs already on the market. Because serine protease could be an excellent target to stop the virus from getting inside the cell, the analyzed complexes are an excellent place to start looking for new drugs to treat COVID-19.

## Data Availability

The original contributions presented in the study are included in the article/Supplementary Material, further inquiries can be directed to the corresponding authors.
