# Supplementary material for: Interaction of copper potential metallodrugs with TMPRSS2: A comparative study of docking tools and its implications on COVID-19
Source: Front Chem. 2023 Jan 26;11:1128859. doi: 10.3389/fchem.2023.1128859 (PMC9909424; doi:10.3389/fchem.2023.1128859)
Supplement: Supplementary file 1 [file DataSheet1.pdf]

*Supplementary Material*

# Interaction of copper potential metallodrugs with TMPRSS2: A comparative study of docking tools and its implications on COVID-19

**Sergio Vazquez-Rodriguez[1], Diego Ramírez-Contreras[1], Lisset Noriega[2,3], Amalia García-García[1,4], Brenda L. Sánchez-Gaytán[1], Francisco J. Melendez[2], María Eugenia Castro[1*], Walter Filgueira de Azevedo Jr.[5], Enrique González-Vergara[1*]**

[1]Centro de Química del Instituto de Ciencias, Benemérita Universidad Autónoma de Puebla, 18 sur y Av. San Claudio, Col. San Manuel, 72570 Puebla, Mexico; sergio.vazquez@alumno.buap.mx (S.V.-R.); diego.ramirezco@alumno.buap.mx (D.R.-C.); brenda.sanchez@viep.com.mx (B.L.S.-G.); mareug.castro@correo.buap.mx (M.E.C)

[2]Laboratorio de Química Teórica. Depto. de Fisicoquímica. Facultad de Ciencias Químicas, Benemérita Universidad Autónoma de Puebla, 22 sur y Av. San Claudio, Col. San Manuel, 72570 Puebla, Mexico; lisset.noriegad@alumno.buap.mx (L.N.); francisco.melendez@correo.buap.mx (F.J.M.)

[3]Centro de Investigación y de Estudios Avanzados del Instituto Politécnico Nacional, Unidad Mérida, Carr. Mérida-Progreso, Loma Bonita, 97205 Mérida, Yuc., Mexico; lisset.noriegad@alumno.buap.mx (L.N.)

[4]Departamento de Química Inorgánica, Facultad de Ciencias, Universidad de Granada, Av. Fuentenueva S/N, 18071 Granada, Spain; amaliagar@ugr.es (A.G.-G.)

[5]Escola de Ciências da Saúde, Pontifícia Universidade Católica do Rio Grande do Sul (PUCRS), Ipiranga Avenue, 6681 Partenon, 90619-900 Porto Alegre, RS, Brazil; walter@azevedolab.net (W.F.)

*** Correspondence:**
Corresponding Author: mareug.castro@correo.buap.mx (M.E.C.); enrique.gonzalez@correo.buap.mx (E.G.-V.); Tel.: +52-2223630626.

**Table S1.** Global reactivity indices of the copper complexes calculated with the mPW1PW91 functional in aqueous solution: vertical ionization potential (*I*), vertical electron affinity (*A*), gap energy ($E_{gap}$), chemical potential (μ), electronegativity (χ), hardness (η), softness (s), and electrophilicity index (ω), all in eV units.

| Ligand | *I* | *A* | $E_{gap}$ | μ | χ | η | s | ω |
|---|---|---|---|---|---|---|---|---|
| [Cu (Bipy)(Arg)]$^{2+}$ | 7.2220 | 3.1239 | 4.0981 | -5.1729 | 5.1729 | 2.0490 | 0.2440 | 6.5297 |
| [Cu (Bipy)(Arg)($H_2O$)]$^{2+}$ | 7.1240 | 2.8463 | 4.2777 | -4.9852 | 4.9852 | 2.1388 | 0.2338 | 5.8097 |
| [Cu (Bipy)(Orn)]$^{2+}$ | 7.2328 | 3.1674 | 4.0654 | -5.2001 | 5.2001 | 2.0327 | 0.2460 | 6.6516 |
| [Cu (Bipy)(Orn)($H_2O$)]$^{2+}$ | 7.1349 | 2.8817 | 4.2532 | -5.0083 | 5.0083 | 2.1266 | 0.2351 | 5.8975 |
| [Cu (Bipy)(Lys)]$^{2+}$ | 7.1757 | 3.1266 | 4.0491 | -5.1512 | 5.1512 | 2.0245 | 0.2470 | 6.5532 |
| [Cu (Bipy)(Lys)($H_2O$)]$^{2+}$ | 7.0669 | 2.9008 | 4.1661 | -4.9838 | 4.9838 | 2.0830 | 0.2400 | 5.9620 |
| [Cu (Bipy)(Citr)]$^{1+}$ | 6.8873 | 3.0804 | 3.8069 | -4.9838 | 4.9838 | 1.9035 | 0.2627 | 6.5245 |
| [Cu (Bipy)(Citr)($H_2O$)]$^{1+}$ | 6.8383 | 2.8763 | 3.9620 | -4.8573 | 4.8573 | 1.9810 | 0.2524 | 5.9548 |
| [Cu (Bipy)(Asn)]$^{1+}$ | 7.1158 | 3.0559 | 4.0600 | -5.0858 | 5.0858 | 2.0300 | 0.2463 | 6.3709 |
| [Cu (Bipy)(Asn)($H_2O$)$_2$]$^{1+}$ | 6.8981 | 2.7293 | 4.1688 | -4.8137 | 4.8137 | 2.0844 | 0.2399 | 5.5584 |
| [Cu (Bipy)(The)]$^{1+}$ | 7.1294 | 3.0967 | 4.0328 | -5.1131 | 5.1131 | 2.0164 | 0.2480 | 6.4828 |
| [Cu (Bipy)(Gln)]$^{1+}$ | 7.1458 | 3.0994 | 4.0464 | -5.1226 | 5.1226 | 2.0232 | 0.2471 | 6.4850 |
| [CAS III-ia]$^{1+}$ | 6.8138 | 2.9661 | 3.8477 | -4.8899 | 4.8899 | 1.9239 | 0.2599 | 6.2144 |
| [CAS III-ia($H_2O$)]$^{1+}$ | 6.6342 | 2.7184 | 3.9157 | -4.6763 | 4.6763 | 1.9579 | 0.2554 | 5.5846 |
| [CAS IX-Gly]$^{1+}$ | 7.1893 | 3.1157 | 4.0736 | -5.1525 | 5.1525 | 2.0368 | 0.2455 | 6.5172 |
| [CAS IX-Gly($H_2O$)]$^{1+}$ | 7.0995 | 2.8354 | 4.2641 | -4.9675 | 4.9675 | 2.1320 | 0.2345 | 5.7869 |
| [CAS IX-Gly($H_2O$)$_2$]$^{1+}$ | 6.9988 | 2.7157 | 4.2831 | -4.8573 | 4.8573 | 2.1416 | 0.2335 | 5.5084 |
